# ATF3 and HNF4A: an oxidative phosphorylation and cholesterol homeostasis-associated diagnostic and therapeutic repurposing framework target for metabolic dysfunction-associated steatohepatitis patients

**DOI:** 10.3389/fmed.2026.1772363

**Published:** 2026-07-07

**Authors:** Guiying Zeng, Qi Zhao, Li Jiang, Dongmei Xie, Lin Du, Mei Yang, Mei Luo, Qian Wang

**Affiliations:** 1 Department of Gastroenterology, Guizhou University of Traditional Chinese Medicine, Guiyang, Guizhou, China; 2 Department of Gastroenterology, Qixingguan District People’s Hospital, Bijie, Guizhou, China; 3 Department of Gastroenterology, Guizhou Second People’s Hospital, Guiyang, Guizhou, China; 4 Department of Gastroenterology, Anshun People’s Hospital, Anshun, Guizhou, China; 5 Qinglong County Center for Disease Control and Prevention, Qianxinan Buyei and Miao Autonomous Prefecture, Qinglong, Guizhou, China

**Keywords:** cholesterol homeostasis, diagnostic biomarkers, non-alcoholic steatohepatitis, oxidative phosphorylation, therapeutic approaches

## Abstract

**Objective:**

Metabolic dysfunction-associated steatohepatitis (MASH) is hepatic steatosis. Oxidative phosphorylation and cholesterol homeostasis (OC) plays a key role in the onset and progression of MASH. Hence, deeper understanding of OC in MASH can shed light on the clinical applications for MASH patients.

**Methods:**

Metabolic dysfunction-associated steatohepatitis hepatic bulk profile (GSE89632) was subjected to GSVA and WGCNA analysis for identification of OC-associated highest correlated gene module and then intersected with OC-associated gene list downloaded from Genecard database for acquisition of OC-related DEGs. Next By integration of another MASH hepatic bulk profile (GSE164760) and machine learning algorithms (RF and Lasso) based on OC-associated DEGs, we identified hub variables. Next, OC-related diagnostic model based on hub variables was constructed on GSE164760 and then examined on the GSE89632 and GSE63067 (MASH bulk dataset). In addition, Consensus clustering was performed for the identification of OC-related molecular subgroups for MASH patient in GSE164760 and heterogeneity of hub variables was examined at MASH single-cell transcriptomic dataset (GSE189600) in temporal and spatial manners. DGIDB database with molecular docking and deep learning algorithm (Drugreflector) were performed for the identification of drug repurposing framework for reversing MASH to healthy status based on GSE164760 and potential agent targeting hub variables. *In vitro* study indicated the expression patterns of hub variables in MASH cell lines compared to normal cell lines.

**Results:**

Activating Transcription Factor 3 and HNF4A were down-regulated and up-regulated expression hub variable associated with MASH pathogenesis, which illustrated satisfied diagnostic performance. ALVERINE and MECAMYLAMINE were potential therapeutic approaches for MASH treatment.

**Conclusion:**

Our study first indicated that OC was associated with MASH onset and progression, which can elaborate predictive and therapeutic potentials for MASH patients. Besides, ATF3 and HNF4A can be considered as OC-associated diagnostic and druggable targets for the treatment of MASH.

## 1 Introduction

Metabolic dysfunction-associated steatohepatitis (MASH) is characterized by hepatic fat deposition and affects approximately 25% of the global population, posing a significant public health challenge as its progression can lead to cirrhosis and hepatocellular carcinoma, resulting in high mortality rates (1, 2). Current treatment options for MASH primarily involve lifestyle modifications and pharmacological interventions; however, these methods have shown limited efficacy (3). Hence, it is essential to understand the intricate signaling pathways involved in MASH for providing additional insights into their treatment. Indeed, the central molecular mechanisms underlying MASH pathogenesis include oxidative phosphorylation and dysregulation of cholesterol homeostasis, ensuing induced inflammatory responses, lipid metabolism disturbances, and cell apoptosis, which contributes to the disease’s pathogenesis (4–6). Novelty, recent studies have highlighted the role of cholesterol homeostasis in MASH progression, revealing that disturbances in this balance can exacerbate the disease phenotype (7). Specifically, alterations in hepatic cholesterol metabolism have been implicated in promoting oxidative stress and inflammation, critical factors in the development of MASH (8, 9). Hence, recent advancements into the integration of oxidative phosphorylation and cholesterol homeostasis (OC) contribute to the additional clinical choices for the improvement of patient clinical outcomes.

**FIGURE 1:**
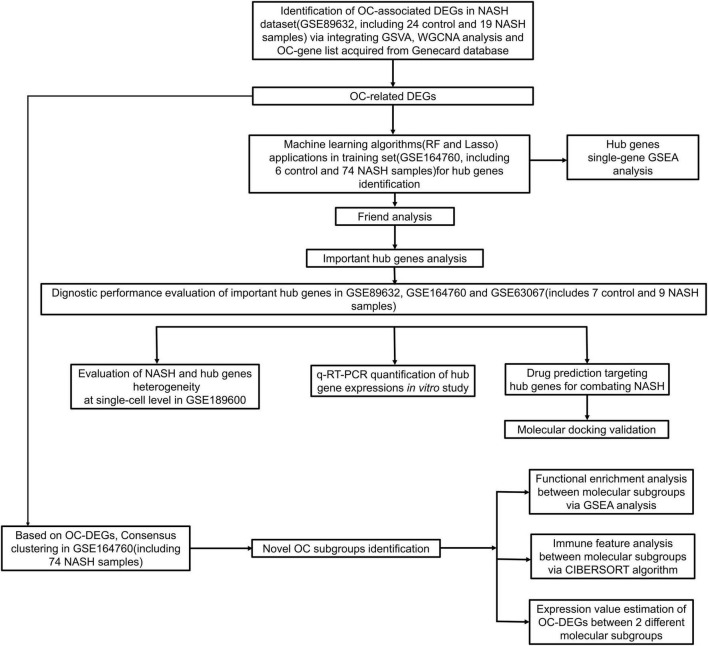
The workflow of this study.

In this study, by utilizing the integrative bioinformatic approaches and multi-omics studies, we identified OC-associated hub variables (ATF3 and HNF4A) in MASH patients and constructed an OC-associated diagnostic model. Indeed, we also deciphered the molecular mechanisms of ATF3 and HNF4A at bulk and single-cell levels in temporal and spatial manners. Furthermore, we also examined the expression patterns of ATF3 and HNF4A *in silico* and *in vitro*. Finally, we discovered that alverine and mecamylamine can be considered as optimal therapeutic framework for the treatment of MASH targeting ATF3 and HNF4A. Besides, based on GSE164760, we also discovered that BRD-K91950687 can be considered as potential therapeutic agent for the treatment of MASH. Our study first revealed that combination of oxidative phosphorylation and cholesterol homeostasis in the pathogenesis of MASH, thereby facilitating the clinical translation in MASH treatment and diagnosis. We describe the workflow of this study in Figure 1.

## 2 Materials and methods

### 2.1 GSVA and weighted gene go-expression network analysis (WGCNA) analysis

We first downloaded the GSE89632 bulk profile (GPL14951, including 24 normal hepatic samples and 19 MASH hepatic samples) from the GEO database via GEOquery package of R, and pending normalization and standardization via Limma package of R (10, 11). Next, we download the hallmark gene set in MSIGDB database and utilize the GSVA package of R for exploring cholesterol homeostasis and oxidative phosphorylation performance across diverse samples (12, 13). Next, based on the performance of OC score, WGCNA is utilized to discern modules of genes exhibiting high correlation, to elucidate the interrelationships among these modules, and to establish their connections with external sample characteristics, thereby facilitating the identification of potential biomarkers or therapeutic targets (14). In the context of our study, WGCNA was implemented through the R package WGCNA to pinpoint modules that show the greatest correlation with OC in patients suffering from MASH (15). Initially, we conducted preprocessing of the sample data to eliminate any outliers (15). Following this, we generated a correlation matrix using the WGCNA package of R software. An optimal soft threshold was selected to transform the correlation matrix into an adjacency matrix, from which a topological overlap matrix (TOM) was derived (15). To categorize genes by exhibiting analogous expression profiles into gene modules, we employed a TOM-based phase dissimilarity metric in conjunction with average linkage hierarchical clustering.(15) The module that displayed the highest correlation with OC were designated as key modules for further analysis. Next, OC-associated greatest correlated module was intersected with OC-related gene list downloaded from Genecard database for acquisition of OC-associated DEGs (16).

### 2.2 Machine learning algorithms and diagnostic value estimation

We downloaded the GSE164760 bulk profile (GPL14951, including 6 normal hepatic samples and 74 MASH hepatic samples) from the GEO database for machine learning model construction via GEOquery package of R, and pending normalization and standardization via Limma package of R (10, 11, 17). The Least Absolute Shrinkage and Selection Operator (LASSO) logistic regression analysis is a sophisticated data mining technique that employs the L1 penalty (lambda) to effectively reduce the coefficients of less significant variables to zero. This process aids in identifying and filtering out the most pertinent variables, thereby facilitating the construction of an optimal classification model (18). Random Forest (RF) analysis is a decision tree-based machine learning method that focuses on evaluating the significance of variables by scoring the importance of each variable (19). In combination of these 2 machine learning models with OC-DEGs, we acquired OC-associated hub indicators for MASH patients in GSE164760. In addition, single-gene GSEA analysis was conducted for deeper understanding of molecular feature of hub indicators via clusterProfiler of R (20). Furthermore, a predictive model was developed utilizing the expression profiles of hub genes in GSE89632, GSE164760 and GSE63067 (MASH bulk profile, including 7 normal hepatic samples and 9 MASH hepatic samples) to estimate MASH risk via ROC, PR curve generated by pROC and ggplot2 packages of R (21). Its reliability was further verified through calibration analysis via rms packages, which compared predicted probabilities with observed clinical results (22).

### 2.3 Consensus clustering

In GSE164760 cohort, the R package ConsensusClusterPlus was utilized to categorize patients with MASH into various molecular subtypes based on OC-associated DEGs (23). The optimal number of clusters was established by examining the relative alteration in the area under the cumulative distribution function (CDF) curve across a spectrum of clustering values. This clustering algorithm was executed multiple times, ultimately revealing two distinct subgroups, designated as C1 and C2 (23). An analysis of immune infiltration was conducted between these two groups using the CIBERSORT algorithm of R (24). Furthermore, a molecular expression analysis was carried out to identify OC-related DEGs between the subgroups, thereby providing deeper insights into the biological diversity present between the molecular subtypes through GSEA analysis based on the hallmark gene set sourced from the MSIGDB database via clusterProfiler package of R (20).

### 2.4 Single-cell transcriptomic analysis

Single-cell RNA sequencing was performed on 3 MASH samples derived from the GSE189600 dataset to examine the cellular makeup and molecular characteristics linked to MASH (25). Quality control (QC) measures were implemented to eliminate substandard cells based on established filtering criteria, which included the number of detected features, RNA counts, and the percentage of mitochondrial gene expression via Seurat package of R (26). Subsequently, the data underwent dimensionality reduction techniques, specifically UMAP and t-SNE, to facilitate the visualization of cell clustering via Seurat package of R (26). Besides, the Findmarker function is available within the Seurat package of R (26). Cell annotations were assigned through the application of the scMayoMap package of R (27). In addition, CellChat and energy metabolism analysis was conducted to explore the cell-cell communication and metabolism patterns present in the MASH samples, thereby offering insights into the signaling interactions among various cell types (28, 29). Following this, the monocle2 package of R was implemented for tracing the cell differentiation and hub gene expression pattern at spatial and temporal manners in targeted cells (30).

### 2.5 Drug prediction and molecular docking

The Drugreflector framework leverages active learning methodologies, integrating transcriptomic data to identify modulators linked to diverse disease phenotypes (31). Using the GSE164760 cohort dataset, we applied Drugreflector to determine the most effective therapeutic agents for mitigating MASH (31). To assess the drug repurposing agent targeting hub gene, we performed DGIDB database enrichment (32). We selected compounds with highest DIGDB score as optimal agent for further analysis. Next, to assess the binding affinity of these optimal agents with the hub genes, molecular docking studies were conducted. This analysis aimed to explore the interactions between the chosen drugs and their corresponding proteins (33). The target protein PDB files (PDB ID: 4IQR for HNF4A and AlphaFold 2 ID: AF-P18847-5-F1-v6 for ATF3) were retrieved from the RCSB PDB and Alphalfold2 database respectively, while the ligand SDF files (Compound CID: 3678 for Alverine and Compound CID: 4032 for Mecamylamine) were obtained from the PubChem database (34–36). Subsequently, molecular docking was performed to estimate the binding affinities between the target proteins and the respective compounds (37). Initially, PyMOL software (Version 2.6.0) was used to remove water molecules and ligands, preserving only the protein backbone (37). Following this, the AutoDock Vina Tool (Version 4.2.6) was employed to identify potential binding sites on the protein surface and to conduct flexible molecular docking (37). This process involved calculating docking scores and binding affinities (Vina score, kcal/mol) for each identified binding site (37). The favorable binding sites were ranked according to their binding energy, with the site displaying the lowest energy selected for further visualization in PyMOL (37). This visualization step clarified the positions of hydrogen bonds involved in ligand binding, as shown in the resulting images (37). The findings were then illustrated in PyMOL to effectively represent the binding modes and hydrogen bonding interactions (37).

### 2.6 Cell lines and culture

The WRL68 cell line (ID: STM-CL-5610) was purchased from STEM RECELL company (CHINA). The WRL-68 cell line is derived from the healthy liver tissue of a 30-years-old female. Following the thawing process and subsequent recovery, WRL68 cells were cultured in high glucose DMEM (Thermo-Fisher U.S.), enriched with 10% fetal bovine serum (FBS, Thermo-Fisher U.S.) and incubated at a temperature of 37 °C in a humidified environment containing 5% carbon dioxide (CO_2_). Once the cells reached a confluence of 75%–85%, they underwent digestion, passaging, and were subsequently cryopreserved. The fatty acid stock solution was created by combining sodium oleate and sodium palmitate (PWL233-1, MeiLunBio, China) at a molar ratio of 2:1. This stock solution was further diluted to a concentration of 1.2 mmol/L to formulate the fatty acid induction solution (PWL233-1, MeiLunBio, China). The WRL68 cells were categorized into two groups: a control group and a MASH model group. Cells in the control group were maintained in complete medium, whereas those in the model group were exposed to the 1.2 mmol/L fatty acid induction solution. After a 24-h intervention period, relevant cellular indicators were assessed in both groups (38).

### 2.7 RNA extraction and q-RT-PCR

Total RNA was extracted using TRIzol reagent (TaKaRa, Beijing, China), followed by an assessment of its concentration, purity, and integrity through a NanoDrop spectrophotometer (Thermo Scientific, Waltham, MA, USA). For the reverse transcription process, 1 μg of the isolated total RNA was combined with HiScript II Q RT SuperMix for qPCR, which included a gDNA wiper, along with a gDNA eraser (Vazyme, Shanghai, China). The concentration, purity, and integrity of the generated cDNA were then evaluated using the previously mentioned NanoDrop spectrophotometer. Quantitative reverse transcription polymerase chain reaction (q-RT-PCR) was conducted utilizing SYBR Green MasterMix (11203ES50, YEASEN, Shanghai, China) and StepOne Software version 2.3 (Applied Biosystems, Carlsbad, CA, USA) across 40 amplification cycles, incorporating three biological replicates for each sample. The analysis of the data was performed using the ΔΔCt (cycle threshold) method, with normalization against the expression levels of the reference gene, GAPDH. The sequences of the primers used in the qRT-PCR experiments are detailed as follows:

ATF3:

F: 5′-CTGGAAAGTGTGAATGCTGAAC-3′

R: 5′-ATTCTGAGCCCGGACAATAC-3′

HNF4A:

F: 5′- -GGTGTCCATACGCATCCTTGAC -3′

R: 5′- AGCCGCTTGATCTTCCCTGGAT–3′

GAPDH

F 5′- GAGAAGGCTGGGGCTCATTT-3′

R 5′- ATGACGAACATGGGGGCATC-3′.

### 2.8 Statistical analysis

For bioinformatics, all statistical analyses were conducted using R software (version 4.2.2). Differences in the proportions of immune-infiltrating cells within the tumor microenvironment were assessed using the Wilcoxon test. To examine the relationships between different variables, Pearson correlation analysis was applied. Statistical significance was defined as a *p*-value or false discovery rate (FDR) below 0.05. Data are reported as mean ± standard deviation (SD), with significance levels denoted as *p < 0.05, **p < 0.01, and ***p < 0.001. For the experimental section, all statistical evaluations were performed using GraphPad Prism (Version 8.0.2), with each experiment including at least three biological replicates. Results are expressed as mean ± SD. Differences between two datasets were analyzed using either two-way ANOVA or Student’s *t*-test, where a *p*-value less than 0.05 was considered statistically significant.

## 3 Results

### 3.1 Identification of OC-associated DEGs for MASH patients

**FIGURE 2:**
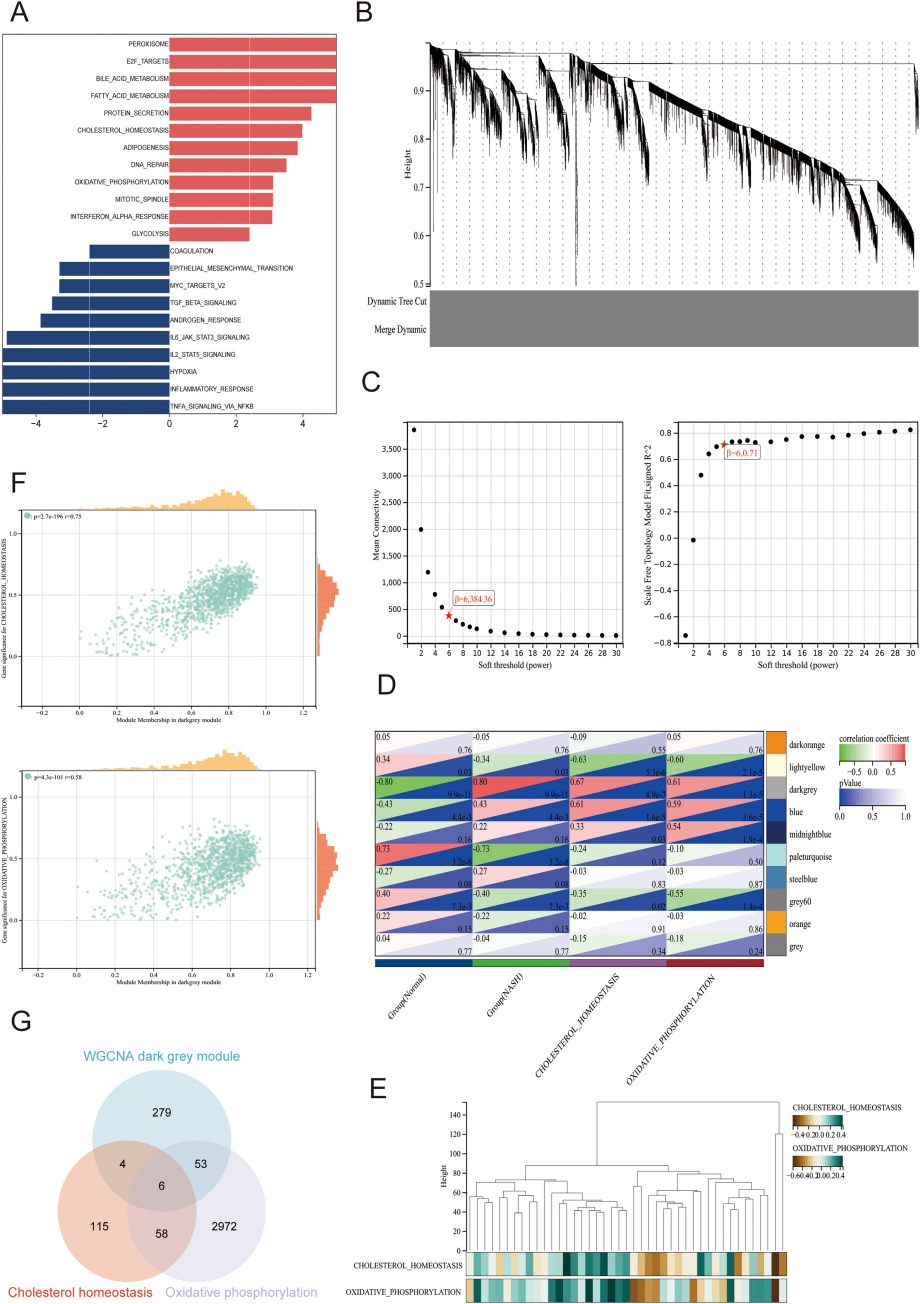
Oxidative phosphorylation and cholesterol homeostasis (OC)-associated DEGs identification for MASH patients. **(A)** GSVA analysis in GSE89632. **(B)** Clustering dendrograms of genes in GSE89632. **(C)** The threshold (β) of WGCNA analysis. **(D)** Color-coded m WGCNA module-trait association heatmap, showing significant. **(E)** Clustering tree of WGCNA analysis. **(F)** Dark gray model gene correlation via WGCNA analysis. **(G)** OC-associated DEGs acquisition.

Firstly, we performed GSVA for estimating various biological and molecular functions in GSE89632, especially OC. The results indicate that OC was activated in MASH patients (Figure 2A). Next, we performed WGCNA to confirm the favorable correlated OC-associated module for MASH patients, and the results indicated Dark gray module shares the highest correlation (Figures 2B–F). OC-associated gene list from Genecard database was intersected with Dark gray module variables for identification of OC-related DEGs (Figure 2G). Finally, 6 OC-associated DEGs was recognized, namely HNF4A, RFC5, HSPA1B, ATF3, SKP2 and COQ10A.

### 3.2 OC-related molecular subgroups identification for MASH patients

**FIGURE 3:**
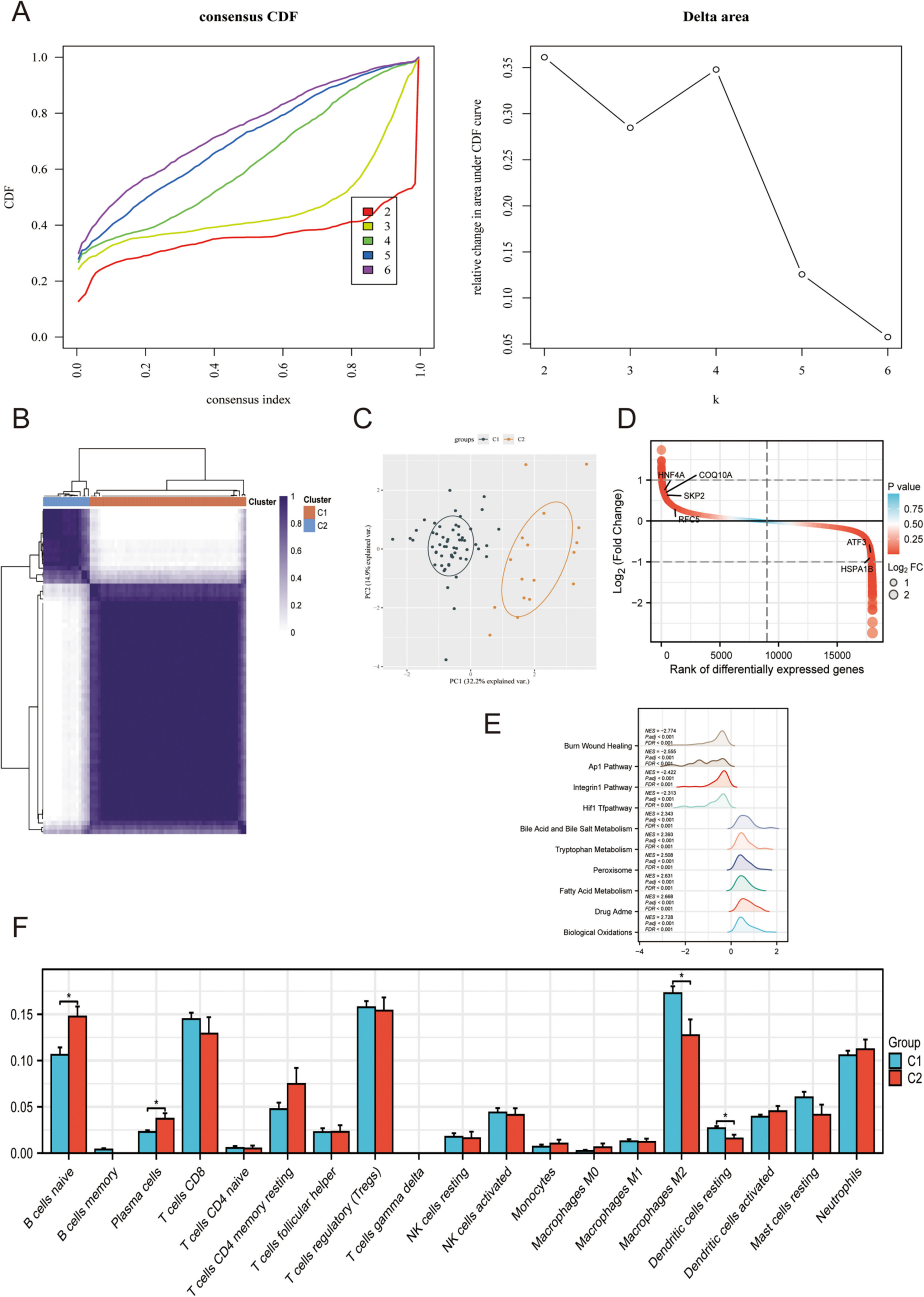
Oxidative phosphorylation and cholesterol homeostasis (OC)-associated subgroups for MASH patients. **(A)** Consensus heatmap. **(B)** CDF curve illustration. **(C)** PCA plot illustration of clustering results. **(D)** Expression heatmap patterns. **(E)** GSEA analysis between C1 and C2. **(F)** Immune infiltration analysis between C1 and C2. *P < 0.05.

In GSE164760 cohort, based on these 6 OC-associated DEGs, we performed consensus clustering for identification of OC-associated subgroups (Figures 3A–C). The results illustrated that there are 2 OC-related subgroups (C1 and C2) (Figures 3B,C). Indeed, COQ10A, SKP2, HSPA1B and RFC5 illustrated increasing expression level, and ATF3 and HNF4A illustrated decreasing expression level in C1 compared to C2 (Figure 3D). Besides, molecular and immune features between C1 and C2 were also assessed (Figures 3E,F).

### 3.3 OC-associated hub indicators identification for MASH patients

**FIGURE 4:**
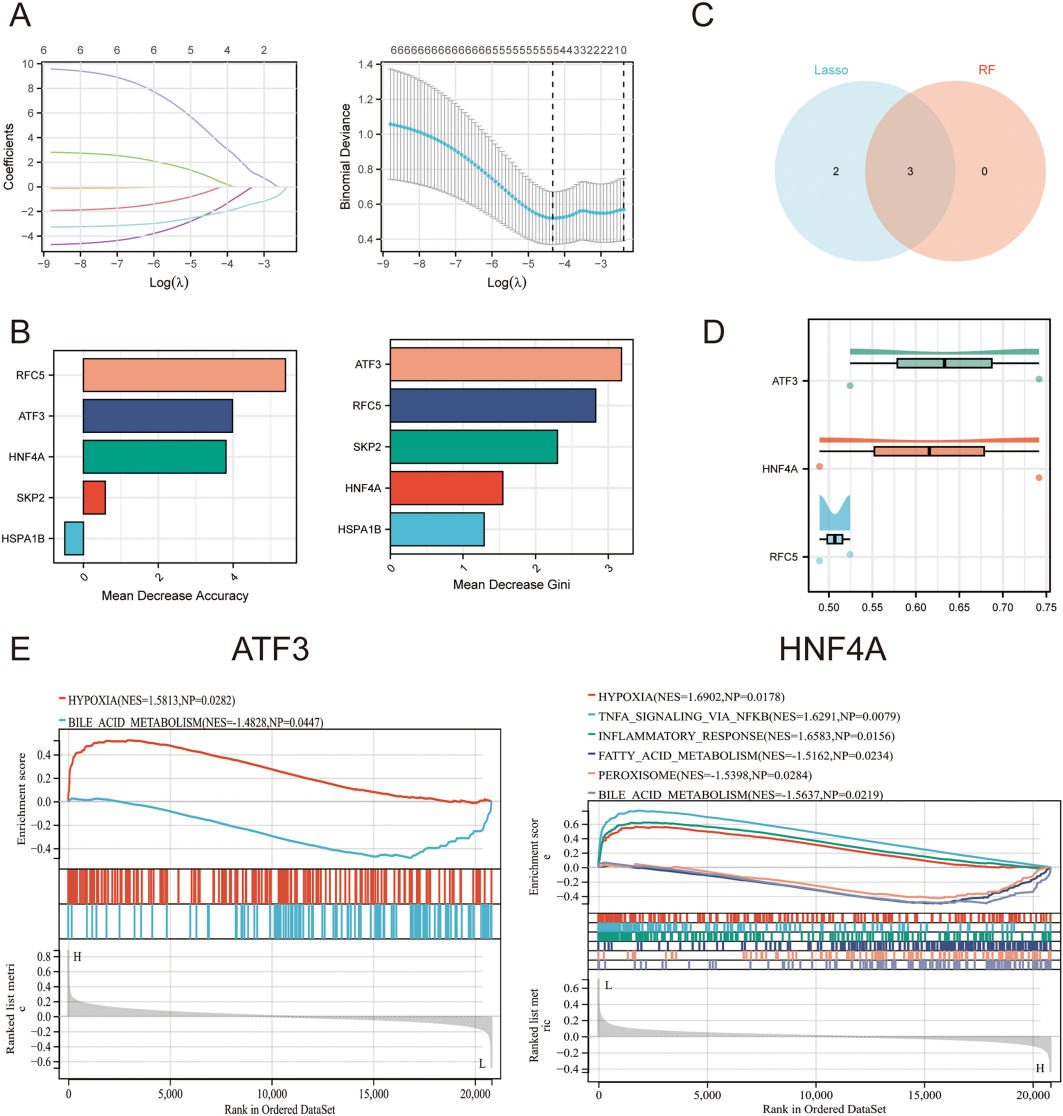
Oxidative phosphorylation and cholesterol homeostasis (OC)-associated hub genes identification for MASH patients. **(A)** Lasso regression results. **(B)** Random forest results. **(C)** Intersection of Lasso and RF results. **(D)** Friend analysis. **(E)** GSEA analysis.

After extracting samples from the GSE164760 cohort, we performed Lasso-Cox regression and Random Forest analysis (Figures 4A,B). Subsequently, 3 hub genes were identified (Figure 4C). Friend analysis revealed that ATF3 and HNF4A were 2 most important variables (Figure 4D). Indeed, ATF3 and HNF4A molecular functions in GSE164760 were estimated (Figure 4E).

### 3.4 OC-associated diagnostic model construction

**FIGURE 5:**
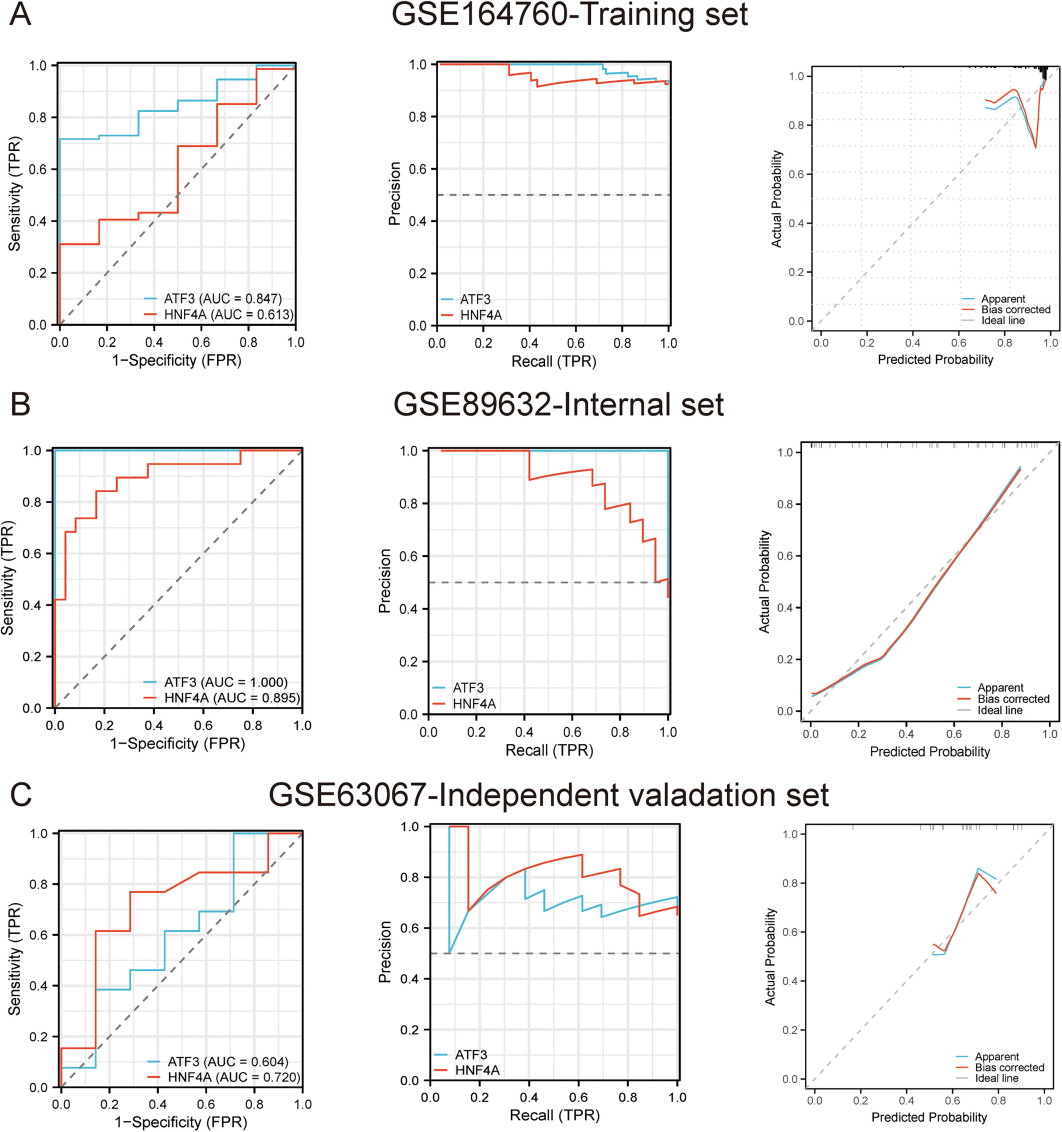
Oxidative phosphorylation and cholesterol homeostasis (OC)-associated diagnostic model construction. **(A)** ROC, PR and calibration analysis in GSE164760. **(B)** ROC, PR and calibration analysis in GSE89632. **(C)** ROC, PR and calibration analysis in GSE63067.

Firstly, we evaluated the diagnostic performance of HNF4A and ATF3 in GSE164760, and then examined both GSE89632 and GSE63067 with ROC, PR and calibration (Figures 5A–C). The results indicated that HNF4A and ATF3 possessed favorable diagnostic efficacy and accuracy.

### 3.5 Analysis of OC-hub indicators at single-cell transcriptomic level

**FIGURE 6:**
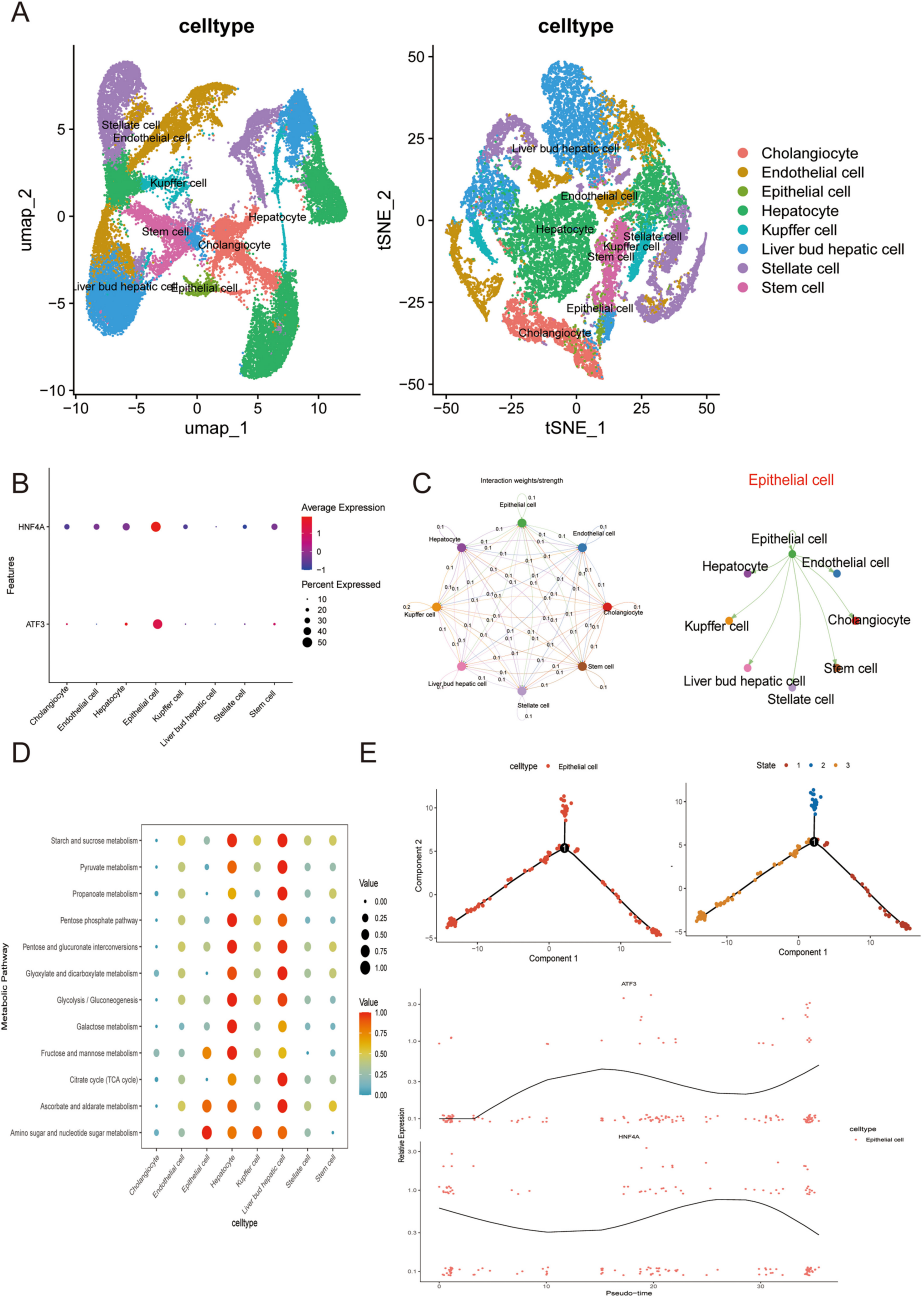
The heterogeneity of HNF4A and ATF3 at MASH single-cell level. **(A)** Single-cell analysis of the distribution of different cell types in MASH. **(B)** Expression of HNF4A and ATF3 among various cell types. **(C)** Cell chat analysis among various cell types. **(D)** Energy metabolism of HNF4A and ATF3 among various cell types. **(E)** Cell trajectory and HNF4A and ATF3 in epithelial cells in temporal and spatial manners.

We assessed the expression profiles of HNF4A and ATF3 at the single-cell resolution in patients with MASH (GSE189600). Utilizing UMAP and t-SNE analytical techniques, we illustrated the distribution of distinct cell populations within the MASH context, which includes (Figure 6A). Furthermore, HNF4A and ATF3 were mainly distributed at epithelial cells and hepatocytes in MASH tissues (Figure 6B). Cell network and energy metabolism analysis underscored the interactions among these cellular entities, thereby reinforcing the fundamental involvement of these cell types in MASH progression (Figures 6C,D). Besides, the cell trajectory of epithelial cell was performed, and the expression of HNF4A and ATF3 in epithelial cells differentiation was also evaluated (Figure 6E).

### 3.6 *In vitro* examination of OC-associated hub indicators expression and targeted drug enrichment

**FIGURE 7:**
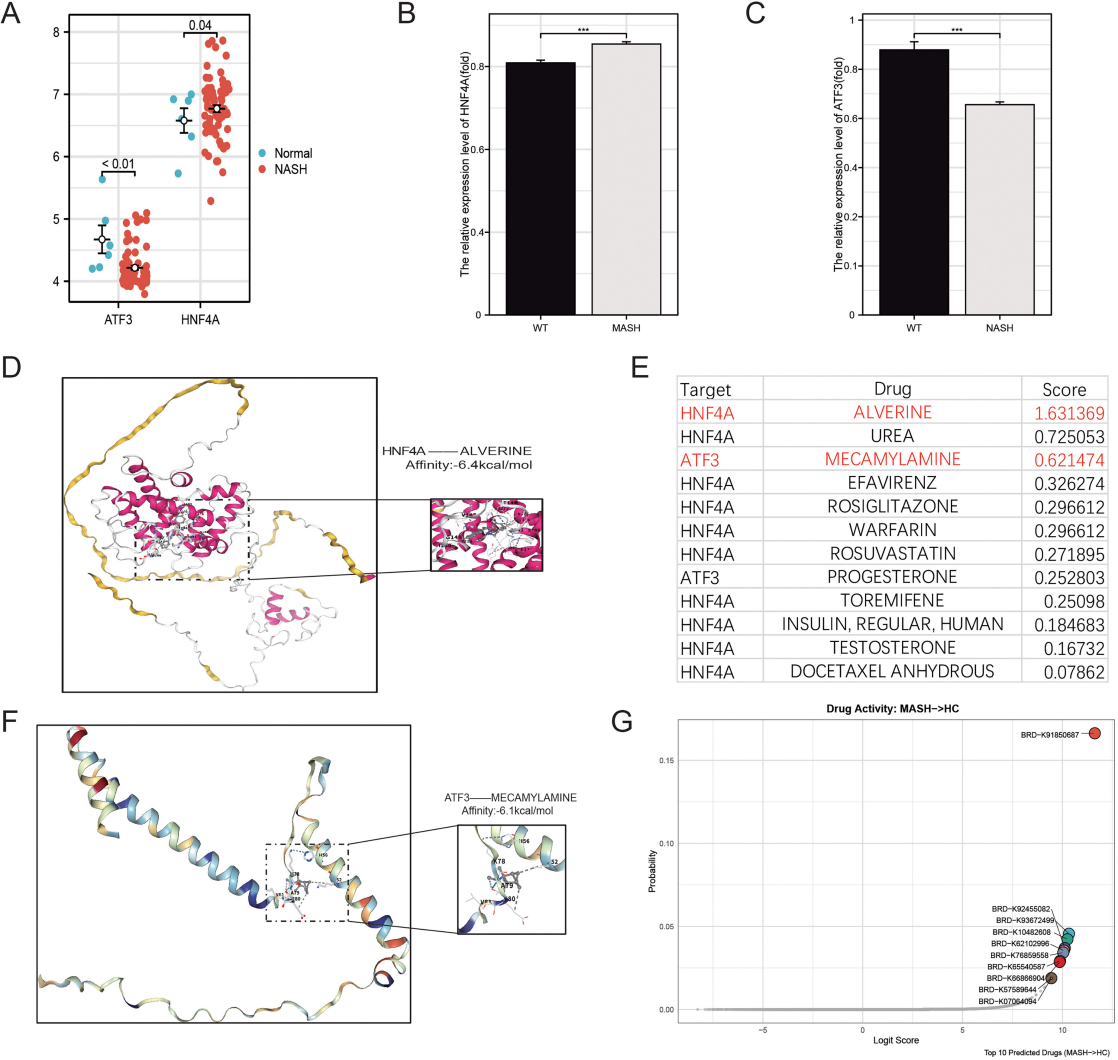
Expression patterns of HNF4A and ATF3 and drug repurposing framework. **(A)** Relative HNF4A and ATF3 *in silico*. **(B)** Relative HNF4A mRNA levels in MASH and control cell groups. **(C)** Relative ATF3 mRNA levels in MASH and control cell groups. **(D)** Molecular docking analysis between HNF4A and ALVERINE. **(E)** DGIDB database enrichment analysis. **(F)** Molecular docking analysis between ATF3 and MECAMYLAMINE. **(G)** Drugreflector enrichment based on GSE164760. ***P < 0.001.

Firstly, we evaluated the expression of HNF4A and ATF3 in GSE164760, and discovered that HNF4A illustrated up-regulated expression patterns and ATF3 illustrated down-regulated expression patterns in MASH patients compared to normal control (Figure 7A). Next, *in vitro* assays indicated that HNF4A illustrated up-regulated expression pattern and ATF3 illustrated down-regulated in MASH-simulated WRL68 compared to normal WRL68 cell lines at mRNA level (Figures 7B,C). Besides, HNF4A and ATF3-oriented drug repurposing frameworks were enriched by MSIGDB database, and ALVERINE with MECAMYLAMINE were considered as potential therapeutic agents with highest DGIDB score by targeting HNF4A and ATF3 respectively (Figure 7E). Next, molecular docking indicates that there are satisfied binding affinity of HNF4A-Alverine complex (−6.4 kcal/mol) and ATF3-Mecamylamine complex (−6.1 kcal/mol), which indicated that these 2 compounds can elaborate therapeutic effects for MASH patients by targeting ATF3 and HNF4A (Figure 7D,F). Besides, based on GSE164760, active learning pipeline (Drugreflector) identified 10 therapeutic agent for the treatment of MASH, and BRD-K91950687 can be considered as optimal one (Figure 7G).

## 4 Discussion and conclusion

Metabolic dysfunction-associated steatohepatitis is a progressive liver disease characterized by inflammation and fibrosis, often leading to cirrhosis and hepatocellular carcinoma (39). Current therapeutic strategies for MASH are limited, primarily focusing on lifestyle modifications and symptomatic management, which underscores the necessity for novel diagnostic and therapeutic approaches (40). In our study, we first unveiled integrated cholesterol homeostasis and oxidative phosphorylation mechanisms in the pathogenesis of MASH, and its corresponding predictive potential for MASH patients via multi-omics and machine learning, which offers novel idea for MASH patient precision medicine. Besides, we also highlighted ATF3 and HNF4A hub pathogenic and therapeutic potentials in MASH. In addition, we also discovered that agents targeting reversing MASH to health status, which provides potential drug synergy strategies for MASH patients.

Dysregulation of cholesterol homeostasis and oxidative phosphorylation have long been considered as 2 major phenotypes involved in MASH pathogenesis (9, 41). To be more specific, abnormal mitochondrial functions, such as excessive fatty acid oxidation and oxidative phosphorylation can lead to the progression of metabolic dysfunction-associated fatty liver disease (MAFLD) to MASH, and the hepatocirrhosis (5). Besides, the dysregulation of cholesterol homeostasis can lead to the abnormal lipid metabolism for MAFLD and MASH patients of which then aggravates the disease (6). Importantly, the cross-talk between oxidative phosphorylation and cholesterol homeostasis has not yet been elucidated. ATF3 (Activating Transcription Factor 3) and HNF4A (Hepatocyte Nuclear Factor 4 Alpha) as key regulatory factors in MASH development mainly distributed at epithelial cells of hepatic tissues, which showed favorable diagnostic performance. For example, hepatic IRF2BP2 can mitigate progression of MAFLD via directly repressing the activity of ATF3 (42). Suppression of ATF3 also can inhibit the ferroptosis of hepatocytes in MASH patients (43). Activating ATF3 is linked to hepatic steatosis for the impairment of glucose homeostasis (44). In the aspect of OC, ATF3 can be considered as regulator for modulating cholesterol homeostasis and oxidative phosphorylation (45, 46). In addition, HNF4A (Hepatocyte Nuclear Factor 4 Alpha), a key regulator of glucose and lipid metabolism, has been widely recognized as pathogenic factor for MAFLD and MASH (47). For instance, HNF4A can inhibit hepatic triglyceride accumulation and fatty acid oxidation, thereby suppressed the progression of NAFLD to NASH (48). Significantly, ATF3 can aggravate diet-induced steatohepatitis via activating HNF4A (49). However, whether HNF4A and ATF3 can regulate integrated cholesterol homeostasis and oxidative phosphorylation has not yet been elucidated.

Overall, by integrating artificial intelligence (AI) pipelines, such as machine learning and deep learning, we identified OC-related predictive and therapeutic model for MASH patients, which provides additional choice for future clinical translation. However, there are still limitations in our study. To be more specific, our study identified up-regulated expression pattern of HNF4A and down-regulated pattern of ATF3 in MASH patient and cell groups. Nevertheless, other studies identified the opposite trend (44, 50). These controversy results highlighted that ATF3 and HNF4A expression patterns and pathogenic roles should be elucidated in a large, multi-center study for verification. Besides, other independent investigations also revealed that ATF3 and HNF4A were mainly distributed in hepatocytes in MASH (51, 52). However, our study revelated that ATF3 and HNF4A were mainly distributed in epithelial cells rather than hepatocytes in MASH. These results indicated that the molecular functions of ATF3 and HNF4A, and corresponding spatial distribution in MASH should be validated in a large-sample size study. Besides, in MASH, ATF3 and HNF4A in regulation of OC and therapeutic agent enriched in our study should be validated in pre-clinical and clinical studies to enhance robustness.

## Data Availability

The original contributions presented in this study are included in this article/Supplementary material, further inquiries can be directed to the corresponding authors.
