# Supplementary material for: ATF3 and HNF4A: an oxidative phosphorylation and cholesterol homeostasis-associated diagnostic and therapeutic repurposing framework target for metabolic dysfunction-associated steatohepatitis patients
Source: Front Med (Lausanne). 2026 Jul 7;13:1772363. doi: 10.3389/fmed.2026.1772363 (PMC13384922; doi:10.3389/fmed.2026.1772363)
Supplement: Supplementary file 1 [file Supplementary_file_1.docx]

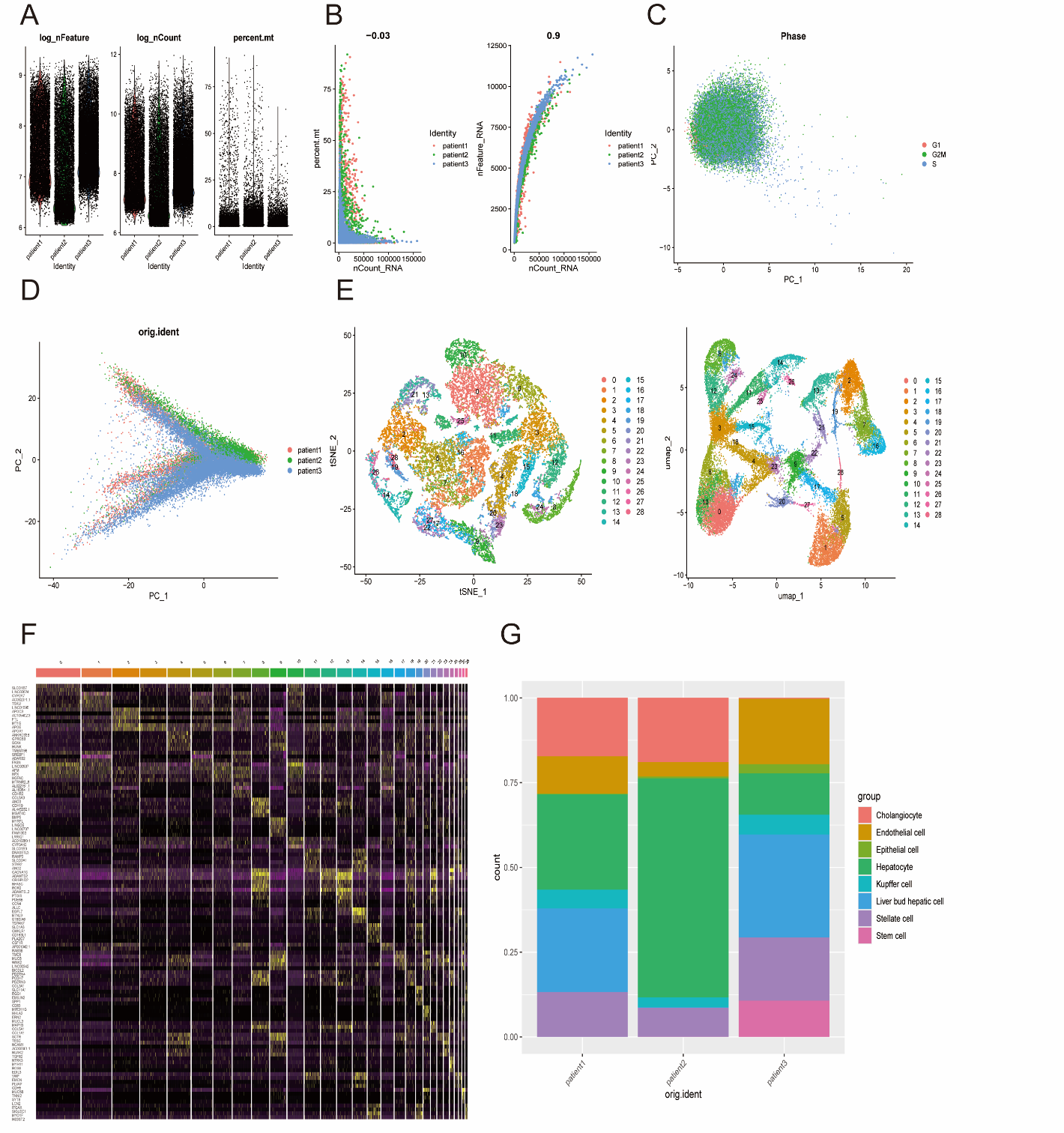


**Figure S1:(A-B)** Violin plot showing the distribution of feature counts, RNA levels, and mitochondrial gene percentages across different cell types. **(C)** PCA plot displaying the distribution of different cell cycle phases (G1, G2M, S) in the NASH dataset. **(D)** PCA plot of sample gene distribution. **(E)** t-SNE and UMAP plot showing the clustering of different cell types in the NASH dataset. **(F)** Heatmap displaying the expression of key genes across different cell types in the NASH dataset. **(G)** Stacked bar chart showing the proportion of cell types in different groups.
